# Supplementary material for: Genomic prediction of alcohol-related morbidity and mortality
Source: Transl Psychiatry. 2020 Jan 21;10:23. doi: 10.1038/s41398-019-0676-2 (PMC7026428; doi:10.1038/s41398-019-0676-2)
Supplement: Supplementary file 1 — SI. Supplementary Information [file 41398_2019_676_MOESM1_ESM.docx]

**SI. Supplementary Information**

[Contributors S1. FinnGen 2](#_Toc19635366)

[Contributors S2. GSCAN Consortium 7](#_Toc19635397)

[Methods S1. Genotyping and imputation 11](#_Toc19635400)

[Fig. S1. Different LDpred PRSs built with varying tuning parameters and respective R-squared values of the linear regression models explaining alcohol consumption with the PRS of alcohol consumption and basic covariates in FINRISK. 13](#_Toc19635401)

[Fig. S2. Cohort and sex-specific alcohol consumption estimate distributions 14](#_Toc19635403)

[Fig. S3. Cohort specific alcohol drinking (g/week) for the deciles of the alcohol consumption PRS 16](#_Toc19635404)

[Table S1. The prospective epidemiological and disease-based cohorts and hospital-based samples in FinnGen Data Freeze 2 18](#_Toc19635405)

[Table S2. Specific conditions and corresponding ICD/ATC codes that were used in the construction of the combinatory alcohol-related morbidities endpoints 19](#_Toc19635406)

[Supplementary References 20](#_Toc19635407)

##

## Contributors S1. FinnGen

# Steering Committee

Aarno Palotie University of Helsinki / FIMM

Mark Daly University of Helsinki / FIMM

## Pharma

Howard Jacob Abbvie

Athena Matakidou Astra Zeneca

Heiko Runz Biogen

Sally John Biogen

Robert Plenge Celgene

Julie Hunkapiller Genentech

Meg Ehm GSK

Dawn Waterworth GSK

Caroline Fox Merck

Anders Malarstig Pfizer

Kathy Klinger Sanofi

Kathy Call Sanofi

## UH & Biobanks

Tomi Mäkelä University of Helsinki /FIMM

Jaakko Kaprio University of Helsinki / FIMM

Petri Virolainen Auria BB / Univ. of Turku /VSSHP

Kari Pulkki Auria BB / Univ. of Turku /VSSHP

Terhi Kilpi THL Biobank (BB) / THL

Markus Perola THL Biobank (BB) / THL

Jukka Partanen Finnish Red Cross Blood Service/FHRB

Anne Pitkäranta HUS/Univ Hosp Districts

Riitta Kaarteenaho Borealis BB/Univ. of Oulu/PPSHP

Seppo Vainio Borealis BB/Univ. of Oulu/PPSHP

Kimmo Savinainen Tampere BB/Univ Tampere/PSHP

Veli-Matti Kosma Eastern Finland BB/UEF/PSSHP

Urho Kujala Central Finland BB /UJy/KSSHP

## Other Experts/ Non-Voting Members

Outi Tuovila Business Finland

Minna Hendolin Business Finland

Raimo Pakkanen Business Finland

# Scientific Committee

## Pharma

Jeff Waring Abbvie

Bridget Riley-Gillis            AbbVie

Athena Matakidou Astra Zeneca

Heiko Runz Biogen

Jimmy Liu Biogen

Shameek Biswas Celgene

Julie Hunkapiller Genentech

Dawn Waterworth GSK

Meg Ehm GSK

Josh Hoffman GSK

Dorothee Diogo Merck

Caroline Fox Merck

Anders Malarstig Pfizer

Catherine Marshall Pfizer

Xinli Hu Pfizer

Kathy Call Sanofi

Kathy Klinger Sanofi

## UH & Biobanks

Samuli Ripatti University of Helsinki / FIMM

Johanna Schleutker Auria BB / Univ. of Turku /VSSHP

Markus Perola THL Biobank (BB) / THL

Tiina Wahlfors Finnish Red Cross Blood Service/FHRB

Olli Carpen HUS/Univ Hosp Districts

Johanna Myllyharju Borealis BB/Univ. of Oulu/PPSHP

Johannes Kettunen Borealis BB/Univ. of Oulu/PPSHP

Reijo Laaksonen Tampere BB/UnivTampere/PSHP

Arto Mannermaa Eastern Finland BB/UEF/PSSHP

Juha Paloneva Central Finland BB /UJy/KSSHP

Urho Kujala Central Finland BB /UJy/KSSHP

## Other Experts/ Non-Voting Members

Outi Tuovila Business Finland

Minna Hendolin Business Finland

Raimo Pakkanen Business Finland

# Clinical Groups

## Neurology Group

Hilkka Soininen LEAD: Kuopio

Valtteri Julkunen Kuopio

Anne Remes Oulu

Reetta Kälviäinen Kuopio

Mikko Hiltunen Kuopio

Jukka Peltola Tampere

Pentti Tienari Helsinki

Juha Rinne Turku

Adam Ziemann AbbVie

Jeffrey Waring AbbVie

Sahar Esmaeeli AbbVie

Nizar Smaoui AbbVie

Anne Lehtonen AbbVie

Susan Eaton Biogen

Heiko Runz Biogen

Sanni Lahdenperä Biogen

Janet van Adelsberg Celgene

Shameek Biswas Celgene

John Michon Genentech

Geoff Kerchner Genentech

Julie Hunkapiller Genentech

Natalie Bowers Genentech

Edmond Teng Genentech

John Eicher Merck

Vinay Mehta Merck

Padhraig Gormley Merck

Kari Linden Pfizer

Christopher Whelan Pfizer

Fanli Xu GSK

David Pulford GSK

## Gastroenterology Group

Martti Färkkilä LEAD:Helsinki

Sampsa Pikkarainen HUS

Airi Jussila Tampere

Timo Blomster Oulu

Mikko Kiviniemi Kuopio

Markku Voutilainen Turku

Bob Georgantas AbbVie

Graham Heap AbbVie

Jeffrey Waring AbbVie

Nizar Smaoui AbbVie

Fedik Rahimov AbbVie

Anne Lehtonen AbbVie

Keith Usiskin Celgene

Tim Lu Genentech

Natalie Bowers Genentech

Danny Oh Genentech

John Michon Genentech

Vinay Mehta Merck

Dermot Reilly Merck

Kirsi Kalpala Pfizer

Melissa Miller Pfizer

Xinli Hu Pfizer

Linda McCarthy GSK

## Rheumatology Group

Kari Eklund LEAD:Helsinki

Antti Palomäki Turku

Pia Isomaki Tampere

Laura Pirilä Turku

Oili Kaipiainen-Seppänen Kuopio

Johanna Huhtakangas Oulu

Bob Georgantas AbbVie

Jeffrey Waring AbbVie

Fedik Rahimov AbbVie

Apinya Lertratanakul AbbVie

Nizar Smaoui AbbVie

Anne Lehtonen AbbVie

David Close AstraZeneca

Marla Hochfeld Celgene

Natalie Bowers Genentech

John Michon Genentech

Dorothee Diogo Merck

Vinay Mehta Merck

Kirsi Kalpala Pfizer

Nan Bing Pfizer

Xinli Hu Pfizer

Jorge Esparza Gordillo GSK

Nina Mars University of Helsinki / FIMM

## Pulmonology Group

Tarja Laitinen LEAD:Tampere

Margit Pelkonen Kuopio

Paula Kauppi Helsinki

Hannu Kankaanranta Tampere

Terttu Harju Oulu

Nizar Smaoui AbbVie

David Close AstraZeneca

Steven Greenberg Celgene

Hubert Chen Genentech

Natalie Bowers Genentech

John Michon Genentech

Vinay Mehta Merck

Jo Betts GSK

Soumitra Ghosh GSK

## Cardiometabolic Diseases Group

Veikko Salomaa Lead: THL

Teemu Niiranen THL

Markus Juonala Turku

Kaj Metsärinne Turku

Mika Kähönen Tampere

Juhani Junttila Oulu

Markku Laakso Kuopio

Jussi Pihlajamäki Kuopio

Juha Sinisalo Helsinki

Marja-Riitta Taskinen Helsinki

Tiinamaija Tuomi Helsinki

Jari Laukkanen Keski-Suomen Keskussairaala

Ben Challis AstraZeneca

Keith Usiskin Celgene

Andrew Peterson Genentech

Julie Hunkapiller Genentech

Natalie Bowers Genentech

John Michon Genentech

Dorothee Diogo Merck

Dermot Reilly Merck

Audrey Chu Merck

Vinay Mehta Merck

Jaakko Parkkinen Pfizer

Melissa Miller Pfizer

Anthony Muslin Sanofi

Dawn Waterworth GSK

## Oncology Group

Heikki Joensuu Lead: Helsinki

Tuomo Meretoja Helsinki

Olli Carpen Helsinki

Lauri Aaltonen Helsinki

Annika Auranen Tampere

Peeter Karihtala Oulu

Saila Kauppila Oulu

Päivi Auvinen Kuopio

Klaus Elenius Turku

Relja Popovic AbbVie

Jeffrey Waring AbbVie

Bridget Riley-Gillis AbbVie

Anne Lehtonen AbbVie

Athena Matakidou AstraZeneca

Jennifer Schutzman Genentech

Julie Hunkapiller Genentech

Natalie Bowers Genentech

John Michon Genentech

Vinay Mehta Merck

Andrey Loboda Merck

Aparna Chhibber Merck

Heli Lehtonen Pfizer

Stefan McDonough Pfizer

Marika Crohns Sanofi

Diptee Kulkarni GSK

## Opthalmology Group

Kai Kaarniranta Lead: Kuopio

Joni Turunen HUS/ Secretary

Terhi Ollila HUS

Sanna Seitsonen HUS

Hannu Uusitalo Tampere

Vesa Aaltonen Turku

Hannele Uusitalo-Järvinen PSHP

Marja Luodonpää Oulu

Nina Hautala Oulu

Heiko Runz Biogen

Erich Strauss Genentech

Natalie Bowers Genentech

Hao Chen Genentech

John Michon Genentech

Anna Podgornaia Merck

Vinay Mehta Merck

Dorothee Diogo Merck

Joshua Hoffman GSK

## Dermatology Group

Kaisa Tasanen Oulu

Laura Huilaja Oulu

Katariina Hannula-Jouppi HUS

Teea Salmi Tampere

Sirkku Peltonen Turku

Leena Koulu Turku

Ilkka Harvima Kuopio

Kirsi Kalpala Pfizer

Ying Wu Pfizer

David Choy Genentech

John Michon Genentech

Nizar Smaoui AbbVie

Fedik Rahimov AbbVie

Anne Lehtonen AbbVie

Dawn Waterworth GSK

# FinnGen Teams

## Administration Team

Anu Jalanko University of Helsinki / FIMM

Risto Kajanne University of Helsinki / FIMM

Ulrike Lyhs University of Helsinki / FIMM

## Communication

Mari Kaunisto University of Helsinki / FIMM

## Analysis Team

Justin Wade Davis Abbvie

Bridget Riley-Gillis Abbvie

Danjuma Quarless Abbvie

Slavé Petrovski Astra Zeneca

Jimmy Liu Biogen

Chia-Yen Chen Biogen

Paola Bronson Biogen

Robert Yang Celgene

Joseph Maranville Celgene

Shameek Biswas Celgene

Diana Chang Genentech

Julie Hunkapiller Genentech

Tushar Bhangale Genentech

Natalie Bowers Genentech

Dorothee Diogo Merck

Emily Holzinger Merck

Padhraig Gormley Merck

Xulong Wang Merck

Xing Chen Pfizer

Åsa Hedman Pfizer

Joshua Hoffman GSK

Clarence Wang Sanofi

Ethan Xu Sanofi

Franck Auge Sanofi

Clement Chatelain Sanofi

Mitja Kurki University of Helsinki / FIMM/ Broad Institute

Samuli Ripatti University of Helsinki / FIMM

Mark Daly University of Helsinki / FIMM

Juha Karjalainen University of Helsinki / FIMM/ Broad Institute

Aki Havulinna University of Helsinki / FIMM

Anu Jalanko University of Helsinki / FIMM

Kimmo Palin University of Helsinki

Priit Palta University of Helsinki / FIMM

Pietro della Briotta Parolo University of Helsinki / FIMM

Wei Zhou Broad Institute

Susanna Lemmelä University of Helsinki / FIMM

Manuel Rivas University of Stanford

Jarmo Harju University of Helsinki / FIMM

Aarno Palotie University of Helsinki / FIMM

Arto Lehisto University of Helsinki / FIMM

Andrea Ganna University of Helsinki / FIMM

Vincent Llorens University of Helsinki / FIMM

Antti Karlsson Auria BB / Univ. of Turku /VSSHP

Kati Kristiansson THL BB / THL

Mikko Arvas Finnish Red Cross Blood Service BB /FHRB

Kati Hyvärinen Finnish Red Cross Blood Service BB /FHRB

Jarmo Ritari Finnish Red Cross Blood Service BB /FHRB

Tiina Wahlfors Finnish Red Cross Blood Service BB /FHRB

Miika Koskinen Helsinki BB/HUS/Univ Hosp Districts

Olli Carpen Helsinki BB/HUS/Univ Hosp Districts

Johannes Kettunen Borealis BB/Univ. of Oulu/PPSHP

Katri Pylkäs Borealis BB/Univ. of Oulu/PPSHP

Marita Kalaoja Borealis BB/Univ. of Oulu/PPSHP

Minna Karjalainen Borealis BB/Univ. of Oulu/PPSHP

Tuomo Mantere Borealis BB/Univ. of Oulu/PPSHP

Eeva Kangasniemi Tampere BB/Univ Tampere/PSHP

Sami Heikkinen Eastern Finland BB/UEF/PSSHP

Arto Mannermaa Eastern Finland BB/UEF/PSSHP

Eija Laakkonen Central Finland BB /UJy/KSSHP

Juha Kononen Central Finland BB /UJy/KSSHP

## Sample Collection Coordination

Anu Loukola Helsinki BB/HUS/Univ Hosp Districts

##

## Sample Logistics

Päivi Laiho THL BB / THL

Tuuli Sistonen THL BB / THL

Essi Kaiharju THL BB / THL

Markku Laukkanen THL BB / THL

Elina Järvensivu THL BB / THL

Sini Lähteenmäki THL BB / THL

Lotta Männikkö THL BB / THL

Regis Wong THL BB / THL

## Registry Data Operations

Kati Kristiansson THL BB / THL

Hannele Mattsson THL BB / THL

Susanna Lemmelä University of Helsinki / FIMM

Tero Hiekkalinna THL BB / THL

Manuel González Jiménez THL BB / THL

##

## Genotyping

Kati Donner University of Helsinki / FIMM

## Sequencing Informatics

Priit Palta University of Helsinki / FIMM

Kalle Pärn University of Helsinki / FIMM

Javier Nunez-Fontarnau University of Helsinki / FIMM

## Data Management and IT Infrastructure

Jarmo Harju University of Helsinki / FIMM

Elina Kilpeläinen University of Helsinki / FIMM

Timo P. Sipilä University of Helsinki / FIMM

Georg Brein University of Helsinki / FIMM

Alexander Dada University of Helsinki / FIMM

Ghazal Awaisa University of Helsinki / FIMM

Anastasia Shcherban University of Helsinki / FIMM

Tuomas Sipilä University of Helsinki / FIMM

## Clinical Endpoint Development

Hannele Laivuori University of Helsinki / FIMM

Aki Havulinna University of Helsinki / FIMM

Susanna Lemmelä University of Helsinki / FIMM

Tuomo Kiiskinen University of Helsinki / FIMM

## Trajectory Team

Tarja Laitinen Tampere University Hospital

Harri Siirtola University of Tampere

Javier Gracia Tabuenca University of Tampere

## Biobank Directors

Lila Kallio Auria Biobank

Sirpa Soini THL Biobank

Jukka Partanen Blood Service Biobank

Kimmo Pitkänen Helsinki Biobank

Seppo Vainio Northern Finland Biobank Borealis

Kimmo Savinainen Tampere Biobank

Veli-Matti Kosma Biobank of Eastern Finland

Teijo Kuopio Central Finland Biobank

##

##

## Contributors S2. GSCAN Consortium

# Affiliations

**Department of Psychology, University of Minnesota Twin Cities, Minneapolis, MN, USA**

Mengzhen Liu, Gargi Datta, Seon-Kyeong Jang, Hannah Young, William G. Iacono, Matt McGue, James J. Lee & Scott Vrieze

**Department of Public Health Sciences, College of Medicine, Pennsylvania State University, Hershey, PA, USA**

Yu Jiang, Fang Chen, Daniel McGuire, Yueh Ling & Dajiang J. Liu

**Institute of Personalized Medicine, College of Medicine, Pennsylvania State University, Hershey, PA, USA**

Yu Jiang, Fang Chen, Daniel McGuire & Dajiang J. Liu

I**nstitute for Behavioral Genetics, University of Colorado Boulder, Boulder, CO, USA**

Robbee Wedow, David M. Brazel, Jason D. Boardman, Marissa A. Ehringer, John K. Hewitt, Christian J. Hopfer, Matthew C. Keller, Kenneth S. Krauter, Matthew B. McQueen, Michael C. Stallings & Jerry A. Stitzel

**Department of Sociology, University of Colorado Boulder, Boulder, CO, USA**

Robbee Wedow & Jason D. Boardman

**Institute of Behavioral Science, University of Colorado Boulder, Boulder, CO, USA**

Robbee Wedow & Jason D. Boardman

**Computer Science and Artificial Intelligence Lab, Massachusetts Institute of Technology, Cambridge, MA, USA**

Yue Li, Jose Davila-Velderrain & Manolis Kellis

**The Broad Institute of MIT and Harvard, Cambridge, MA, USA**

Yue Li, Jose Davila-Velderrain, Tõnu Esko & Manolis Kellis

**Department of Molecular, Cellular, and Developmental Biology, University of Colorado Boulder, Boulder, CO, USA**

David M. Brazel, Yueh Ling & Kenneth S. Krauter

**Interdisciplinary Quantitative Biology Graduate Group, University of Colorado Boulder, Boulder, CO, USA**

David M. Brazel

**23andMe, Inc., Mountain View, CA, USA**

Chao Tian, Michelle Agee, Babak Alipanahi, Adam Auton, Robert K. Bell, Katarzyna Bryc, Sarah L. Elson, Pierre Fontanillas, Nicholas A. Furlotte, David A. Hinds, Bethann S. Hromatka, Karen E. Huber, Aaron Kleinman, Nadia K. Litterman, Matthew H. McIntyre, Joanna L. Mountain, Carrie A. M. Northover, J. Fah Sathirapongsasuti, Olga V. Sazonova, Janie F. Shelton, Suyash Shringarpure, Chao Tian, Joyce Y. Tung, Vladimir Vacic, Catherine H. Wilson, Steven J. Pitts & David A. Hinds

**Quantitative Biomedical Research Center, Department of Clinical Sciences, University of Texas** Southwestern Medical Center, Dallas, TX, USA

Xiaowei Zhan

**Center for the Genetics of Host Defense, Department of Clinical Sciences, University of Texas Southwestern Medical Center, Dallas, TX, USA**

Xiaowei Zhan

**Division of Research, Kaiser Permanente Northern California, Oakland, CA, USA**

Hélène Choquet, Khanh K. Thai, Constance Weisner, Jie Yin & Eric Jorgenson

**Department of Psychiatry, Virginia Institute for Psychiatric and Behavioral Genetics, Virginia Commonwealth University, Richmond, VA, USA**

Anna R. Docherty & Nathan A. Gillespie

**Department of Psychiatry and Human Genetics, University of Utah, Salt Lake City, UT, USA**

Anna R. Docherty

**Survey Research Center, Institute for Social Research, University of Michigan, Ann Arbor, MI, USA**

Jessica D. Faul, Jennifer A. Smith & David R. Weir

**Department of Biostatistics, Center for Statistical Genetics, University of Michigan, Ann Arbor, MI, USA**

Johanna R. Foerster, Lars G. Fritsche, Anita Pandit, Gregory J. M. Zajac, Michael Boehnke & Gonçalo Abecasis

**K.G. Jebsen Center for Genetic Epidemiology, Department of Public Health and Nursing, Norwegian University of Science and Technology, Trondheim, Norway**

Anne Heidi Skogholt, Maiken Elvestad Gabrielsen, Maiken Elvestad Gabrielsen, Anne Heidi Skogholt & Kristian Hveem

**Genetic Epidemiology, QIMR Berghofer Medical Research Institute, Brisbane, Queensland, Australia**

Scott D. Gordon, Nathan A. Gillespie, Nicholas G. Martin & John B. Whitfield

**Division of Public Health Sciences, Fred Hutchinson Cancer Research Center, Seattle, WA, USA**

Jeffrey Haessler, Chu Chen, Charles Kooperberg, Ulrike Peters & Alexander P. Reiner

**Department of Biological Psychology, Vrije Universiteit Amsterdam, Amsterdam, the Netherlands**

Jouke-Jan Hottenga, Gonneke Willemsen & Dorret I. Boomsma

**Program in Genetic Epidemiology and Statistical Genetics, Harvard T.H. Chan School of Public Health, Boston, MA, USA**

Hongyan Huang, Constance Turman, David J. Hunter & Peter Kraft

**Department of Epidemiology, Harvard T.H. Chan School of Public Health, Boston, MA, USA**

Hongyan Huang, Constance Turman, David J. Hunter, Peter Kraft & Eric Rimm

**Department of Complex Trait Genetics, Center for Neurogenomics and Cognitive Research, Vrije Universiteit Amsterdam, Amsterdam, the Netherlands**

Philip R. Jansen, Tinca J. C. Polderman & Danielle Posthuma

**Department of Child and Adolescent Psychiatry, Erasmus MC Rotterdam, Rotterdam, the Netherlands**

Philip R. Jansen

**Estonian Genome Center, University of Tartu, Tartu, Estonia**

Reedik Mägi, Tõnu Esko, Toomas Haller & Andres Metspalu

**Laboratory for Statistical Analysis, RIKEN Center for Integrative Medical Sciences, Yokohama City, Japan**

Nana Matoba, Yoichiro Kamatani & Yukinori Okada

**Department of Population Health Science, Bristol Medical School, Oakfield Grove, Bristol, UK**

George McMahon, Amy E. Taylor & Luisa Zuccolo

**Istituto di Ricerca Genetica e Biomedica, Consiglio Nazionale delle Ricerche, Monserrato, Italy**

Antonella Mulas, Valeria Orrù, Francesco Cucca & Edoardo Fiorillo

**Institute for Molecular Medicine Finland (FIMM), University of Helsinki, Helsinki, Finland**

Teemu Palviainen, Anu Loukola & Jaakko Kaprio

**deCODE Genetics/Amgen, Inc., Reykjavik, Iceland**

Gunnar W. Reginsson, Gyda Bjornsdottir, Daniel F. Gudbjartsson, Hreinn Stefansson, Kari Stefansson & Thorgeir E. Thorgeirsson

**Department of Epidemiology, University of Michigan, Ann Arbor, MI, USA**

Jennifer A. Smith, Wei Zhao & Sharon L. R. Kardia

**Department of Epidemiology, University of Colorado Anschutz Medical Campus, Aurora, CO, USA**

Kendra A. Young & John E. Hokanson

**Department of Computational Medicine and Bioinformatics, University of Michigan, Ann Arbor, MI, USA**

Wei Zhou & Cristen J. Willer

**Avera Institute for Human Genetics, Sioux Falls, SD, USA**

Gareth E. Davies

**Department of Family Medicine and Community Health, Alpert Medical School, Brown University, Providence, RI, USA**

Charles B. Eaton

**Department of Integrative Physiology, University of Colorado Boulder, Boulder, CO, USA**

Marissa A. Ehringer, Matthew B. McQueen & Jerry A. Stitzel

**School of Engineering and Natural Sciences, University of Iceland, Reykjavik, Iceland**

Daniel F. Gudbjartsson

**Department of Sociology, University of North Carolina at Chapel Hill, Chapel Hill, NC, USA**

Kathleen Mullan Harris

**Carolina Population Center, University of North Carolina at Chapel Hill, Chapel Hill, NC, USA**

Kathleen Mullan Harris

**Department of Psychiatry, Washington University in St. Louis, St. Louis, MO, USA**

Andrew C. Heath & Pamela A. F. Madden

**Department of Psychology and Neuroscience, University of Colorado Boulder, Boulder, CO, USA**

John K. Hewitt, Matthew C. Keller & Michael C. Stallings

**Brain and Mind Centre, University of Sydney, Sydney, New South Wales, Australia**

Ian B. Hickie

**Department of Psychiatry, University of Colorado Anschutz Medical Campus, Aurora, CO, USA**

Christian J. Hopfer

**Nuffield Department of Population Health, University of Oxford, Oxford, UK**

David J. Hunter

**Fellows Program, RTI International, Research Triangle Park, NC, USA**

Eric O. Johnson

**Department of Biostatistics, Harvard T.H. Chan School of Public Health, Boston, MA, USA**

Peter Kraft

**Department of Internal Medicine, Institute of Clinical Medicine, University of Eastern Finland,** **Kuopio, Finland**

Markku Laakso & Alena Stančáková

**Department of Medicine, Kuopio University Hospital, Kuopio, Finland**

Markku Laakso

**Psychiatric Genetics, QIMR Berghofer Medical Research Institute, Brisbane, Queensland, Australia**

Penelope A. Lind & Sarah E. Medland

**Department of Biostatistics and Bioinformatics, University of Colorado Anschutz Medical Campus, Aurora, CO, USA**

Sharon M. Lutz

**Department of Genetics, University of North Carolina at Chapel Hill, Chapel Hill, NC, USA**

Karen L. Mohlke

**Department of Internal Medicine, Division of Cardiovascular Medicine, University of Michigan, Ann Arbor, MI, USA**

Jonas B. Nielsen & Cristen J. Willer

**Department of Statistical Genetics, Osaka University Graduate School of Medicine, Suita, Japan**

Yukinori Okada

**Department of Epidemiology, University of Washington, Seattle, WA, USA**

Ulrike Peters & Alexander P. Reiner

**Department of Clinical Genetics, VU Medical Centre Amsterdam, Amsterdam, the Netherlands**

Danielle Posthuma

**Department of Psychiatry, Washington University School of Medicine, St. Louis, MO, USA**

John P. Rice & Laura J. Bierut

**Department of Nutrition, Harvard T.H. Chan School of Public Health, Boston, MA, USA**

Eric Rimm

**Department of Psychological and Brain Sciences, Indiana University, Bloomington, IN, USA**

Richard J. Rose

**SAA—National Center of Addiction Medicine, Vogur Hospital, Reykjavik, Iceland**

Valgerdur Runarsdottir & Thorarinn Tyrfingsson

**Department of Medicine, Vanderbilt University, Nashville, TN, USA**

Hilary A. Tindle

**Department of Psychiatry, University of California, San Diego, San Diego, CA, USA**

Tamara L. Wall

**FORMI and Department of Neurology, Oslo University Hospital, Oslo, Norway**

Amy Mitchell, Bendik S. Winsvold, John-Anker Zwart, Linda M. Pedersen, Marianne Bakke Johnsen, Sigrid Børte, Synne Øien Stensland & Bendik Slagsvold Winsvold

**MRC Integrative Epidemiology Unit, University of Bristol, Bristol, UK**

Luisa Zuccolo & Marcus R. Munafò

**HUNT Research Centre, Department of Public Health and Nursing, Norwegian University of Science and Technology, Levanger, Norway**

Kristian Hveem

**Department of Medicine, Levanger Hospital, Nord-Trøndelag Hospital Trust, Levanger, Norway**

Kristian Hveem

**UK Centre for Tobacco and Alcohol Studies, School of Psychological Science, University of Bristol,** **Bristol, UK**

Marcus R. Munafò

**Department of Genetics, Washington University School of Medicine, St. Louis, MO, USA**

Nancy L. Saccone

**Department of Human Genetics, University of Michigan, Ann Arbor, MI, USA**

Cristen J. Willer

**Department of Preventative Medicine, Northwestern University Feinberg School of Medicine,** **Chicago, IL, USA**

Marilyn C. Cornelis

**Department of Medicine, Stanford University School of Medicine, Stanford, CA, USA**

Sean P. David

**Department of Public Health, University of Helsinki, Helsinki, Finland**

Jaakko Kaprio

**Faculty of Medicine, University of Iceland, Reykjavik, Iceland**

Kari Stefansson

**Department of Neurology, Oslo University Hospital, Oslo, Norway**

Bendik S. Winsvold & John-Anker Zwart

**Department of Health Promotion, Norwegian Institute of Public Health, Bergen, Norway**

Børge Sivertsen

**Department of Mental Health, Faculty of Medicine and Health Sciences, Norwegian University of Science and Technology, Trondheim, Norway**

Børge Sivertsen, Eystein Stordal, Gunnar Morken, Håvard Kallestad, Marit Sæbø Indredavik, Ole Kristian Drange & Ottar Bjerkeset

**Department of Research and Innovation, Helse-Fonna HF, Haugesund, Norway**

Børge Sivertsen & Ingrid Heuch

**Department of Psychiatry, Hospital Namsos, Nord-Trøndelag Health Trust, Namsos, Norway**

Eystein Stordal

**Division of Mental Health Care, St. Olavs Hospital, Trondheim University Hospital, Trondheim, Norway**

Gunnar Morken, Håvard Kallestad & Ole Kristian Drange

**Institute of Clinical Medicine, University of Oslo, Oslo, Norway**

John-Anker Zwart, Marianne Bakke Johnsen & Sigrid Børte

**Department of Psychiatry, Nord-Trøndelag Hospital Trust, Levanger Hospital, Levanger, Norway**

Katrine Kveli Fjukstad

**Department of Laboratory Medicine, Children’s and Women’s Health, Norwegian University of Science and Technology, Trondheim, Norway**

Katrine Kveli Fjukstad

**Regional Centre for Child and Youth Mental Health and Child Welfare, Department of Mental Health, Faculty of Medicine and Health Sciences, Norwegian University of Science and Technology, Trondheim, Norway**

Marit Skrove & Marit Sæbø Indredavik

**Faculty of Nursing and Health Sciences, Nord University, Levanger, Norway**

Ottar Bjerkeset

**Norwegian Centre for Violence and Traumatic Stress Studies, Oslo, Norway**

Synne Øien Stensland

# Consortia

**23andMe Research Team**

Michelle Agee, Babak Alipanahi, Adam Auton, Robert K. Bell, Katarzyna Bryc, Sarah L. Elson, Pierre Fontanillas, Nicholas A. Furlotte, David A. Hinds, Bethann S. Hromatka, Karen E. Huber, Aaron Kleinman, Nadia K. Litterman, Matthew H. McIntyre, Joanna L. Mountain, Carrie A. M. Northover, J. Fah Sathirapongsasuti, Olga V. Sazonova, Janie F. Shelton, Suyash Shringarpure, Chao Tian, Joyce Y. Tung, Vladimir Vacic, Catherine H. Wilson & Steven J. Pitts

**HUNT All-In Psychiatry**

Amy Mitchell, Anne Heidi Skogholt, Bendik S. Winsvold, Børge Sivertsen, Eystein Stordal, Gunnar Morken, Håvard Kallestad, Ingrid Heuch, John-Anker Zwart, Katrine Kveli Fjukstad, Linda M. Pedersen, Maiken Elvestad Gabrielsen, Marianne Bakke Johnsen, Marit Skrove, Marit Sæbø Indredavik, Ole Kristian Drange, Ottar Bjerkeset, Sigrid Børte & Synne Øien Stensland

## Methods S1. Genotyping and imputation

FinnGen samples were genotyped with Illumina and Affymetrix arrays (Thermo Fisher

Scientific, Santa Clara, CA, USA). Genotype calls were made with GenCall and zCall algorithms for

Illumina and AxiomGT1 algorithm for Affymetrix chip genotyping data. Genotyping data produced

with previous chip platforms were lifted over to build version 38 (GRCh38/hg38) following the

protocol described here: dx.doi.org/10.17504/protocols.io.nqtddwn. Samples with sex discrepancies, high genotype missingness (>5%), excess heterozygosity (+-4SD) and non-Finnish ancestry were removed. Variants with high missingness (>2%), deviation from HWE (P <1e-6) and low minor allele count (MAC<3) were removed. Pre-phasing of genotyped data was performed with Eagle 2.3.5 (https://data.broadinstitute.org/alkesgroup/Eagle/) with the default parameters, except the number of conditioning haplotypes was set to 20,000. Imputation was carried out by using the populationspecific SISu v3 imputation reference panel with Beagle 4.1 (version 08Jun17.d8b, https://faculty.washington.edu/browning/beagle/b4_1.html) as described in the following protocol: dx.doi.org/10.17504/protocols.io.nmndc5e. SISu v3 imputation reference panel was developed using the high-coverage (25-30x) whole genome sequencing data generated at the Broad Institute of MIT and Harvard and at the McDonnell Genome Institute at Washington University; and jointly processed at the Broad Institute. Variant callset was produced with GATK HaplotypeCaller algorithm by following GATK best-practices for variant calling. Genotype-, sample- and variant-wise QC was applied in an iterative manner by using the Hail framework v0.1 (https://github.com/hail-is/hail). The resulting high-quality WGS data for 3,775 individuals were phased with Eagle 2.3.5 as described above. Post-imputation quality control involved excluding variants with INFO score < 0.7.

The FINRISK and Health 2000 samples were genotyped using Illumina CoreExome, OMNIExpress, and 610K arrays. Individuals with non-European ancestry or obscure sex were excluded. Quality control (QC) before phasing and imputation excluded variants with missingness >5%, call rate <95%, minor allele count (MAC) <3 (if Zcalled) or MAC <10 (if called using Illumina GenCal), INFO <0.8, minor allele frequency <0.001%, Hardy-Weinberg equilibrium p-value <1*10-10, and heterozygosity exceeding ±4 standard deviations. The QC was performed on simultaneously on all data. Prior to imputation, the haplotypes were estimated using SHAPEIT2 [1]. Imputation was done with IMPUTE2 [2] using high-coverage, population-specific reference panels of 2690 whole-genome and 5093 whole-exome sequences.

Genotyping of the Twin Cohort was done using Illumina Human610-Quad v1.0 B, Human670-QuadCustom v1.0 A, Illumina HumanCoreExome- (12 v1.0 A, 12 v1.1 A, 24 v1.0 A, 24 v1.1 A, 24 v1.2 A) and Affymetrix FinnGen Axiom arrays. The algorithm for genotype calling were Illumina’s GenCall for all HumanCoreExome chip genotypes, Illuminus for 610k & 670k chip genotypes and AxiomGT1 for Affymetrix chip genotypes. Genotype quality control were done in three batches (batch1: 610k+670k, batch2: HumanCoreExome and batch3: Affymetrix chip genotypes) with removing variants with call rate below 97,5% (batch1 and batch3) and 95% (batch2), removing samples with call rate below 98% (batch1) or 95% (batch2 and batch3), removing variants with its minor allele frequency below 1% and Hardy-Weinberg Equilibrium p-value lower than 1e-06. Also, samples from all batches with heterozygosity test method-of-moments F coefficient estimate value below -0.03 or higher than 0.05 (batch1 and batch2) or ±4SD from the mean (batch3) were removed along with the samples which failed sex check or were among the MDS principal component analysis outliers. Total amount of genotyped autosomal variants after QC were 475526 (batch1), 239894 (batch2) and 388673 (batch3). We then performed pre-phasing using Eagle v2.3 [3] and imputation with Minimac3 v2.0.1 using University of Michigan Imputation Server [4]. Genotypes of all batches were imputed to Haplotype Reference Consortium release 1.1 reference panel [5].

## Fig. S1. Different LDpred PRSs built with varying tuning parameters and respective R-squared values of the linear regression models explaining alcohol consumption with the PRS of alcohol consumption and basic covariates in FINRISK.

##
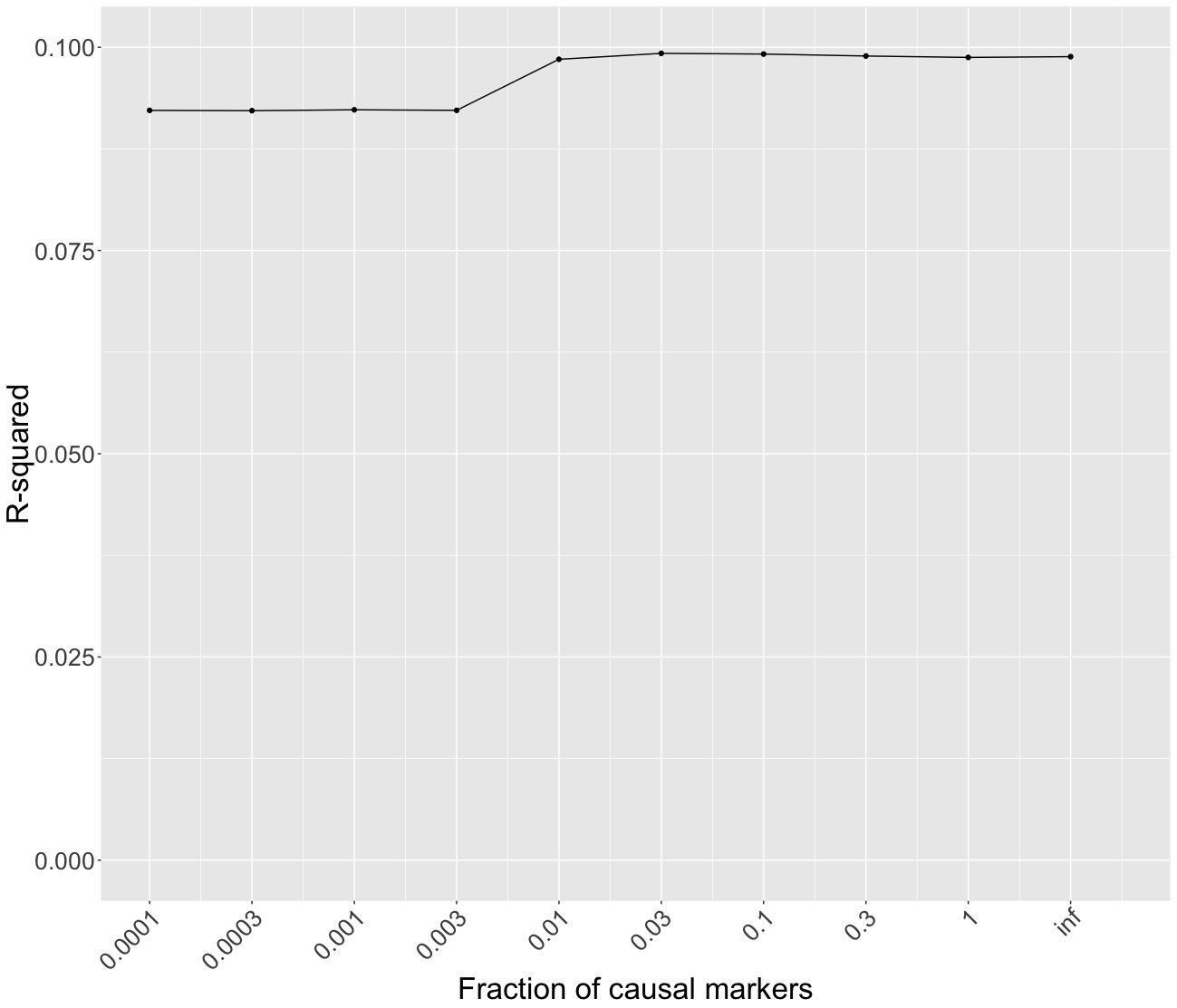


Fig. S2. Cohort and sex-specific alcohol consumption estimate distributions

**a) FINRISK (the smaller inserted graph in the right upper corner of the picture zooms into lower volumes of alcohol consumption for better resolution)**


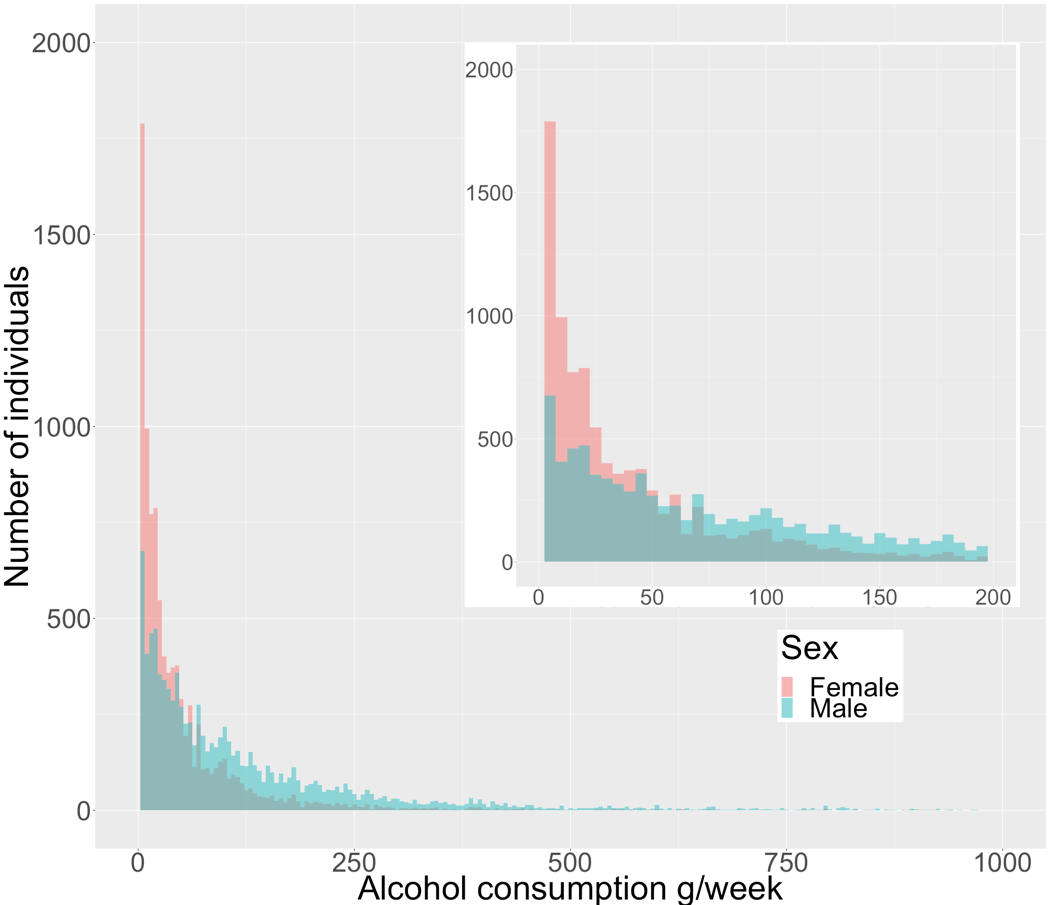


**b)** **Health 2000**

**
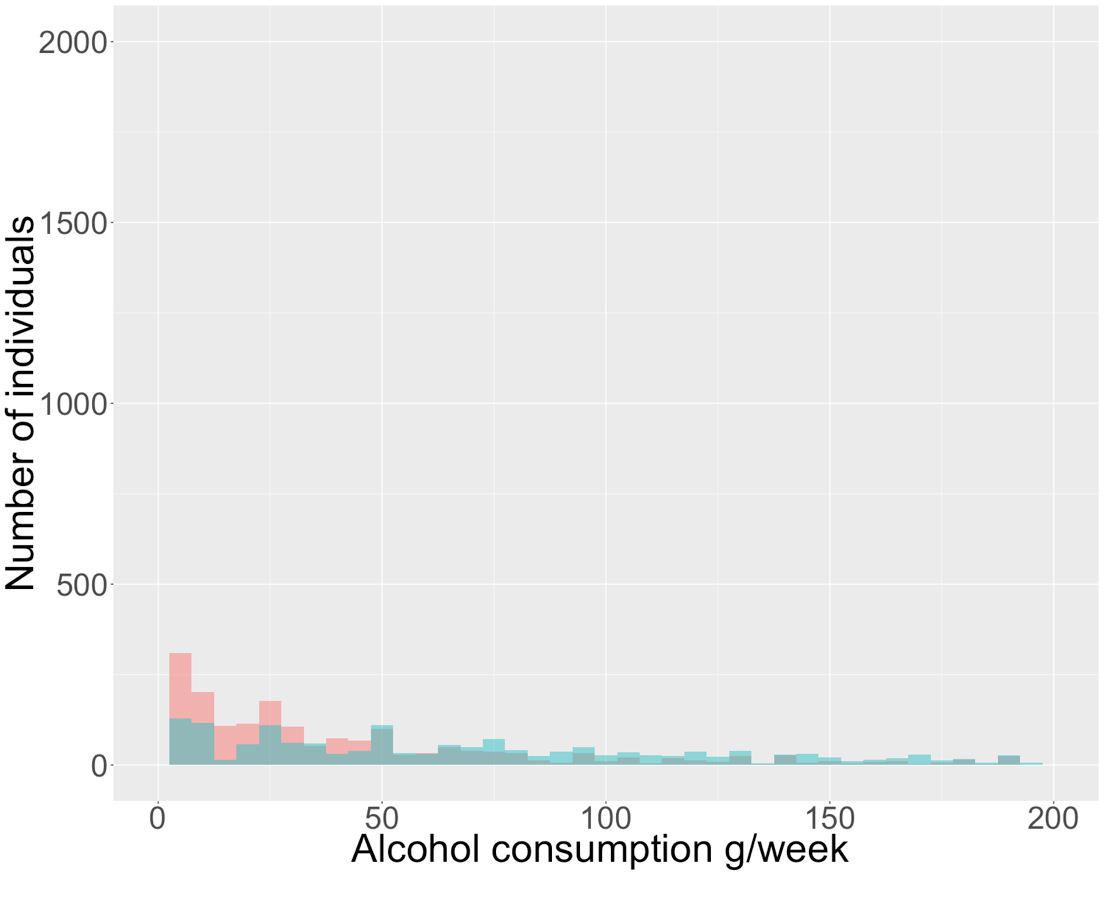
**

**c) Twin Cohort**

**
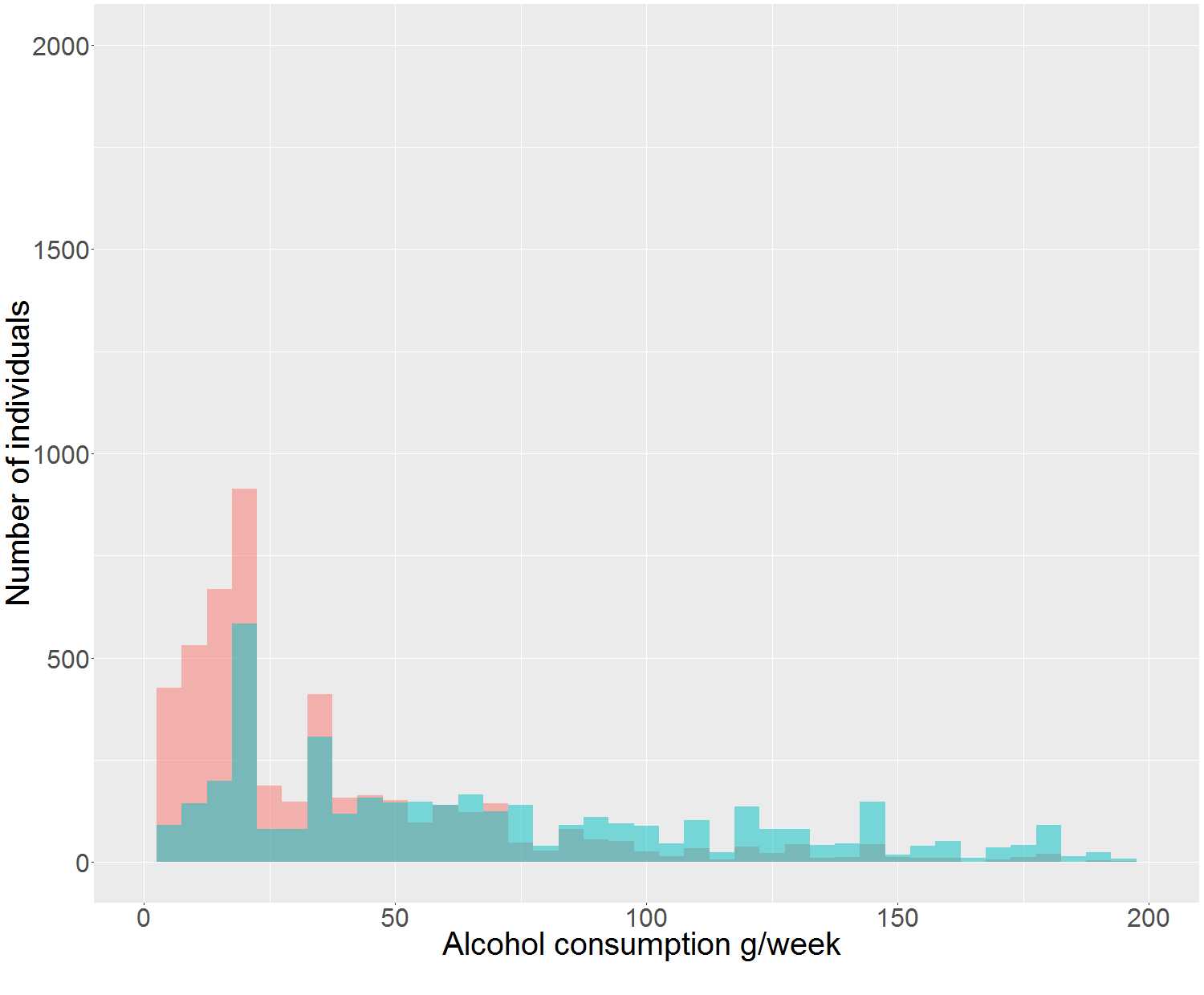
**

Fig. S3. Cohort specific alcohol drinking (g/week) for the deciles of the alcohol consumption PRS *Shown for males and females with 95% confidence interval error bars*

**a) FINRISK**

*
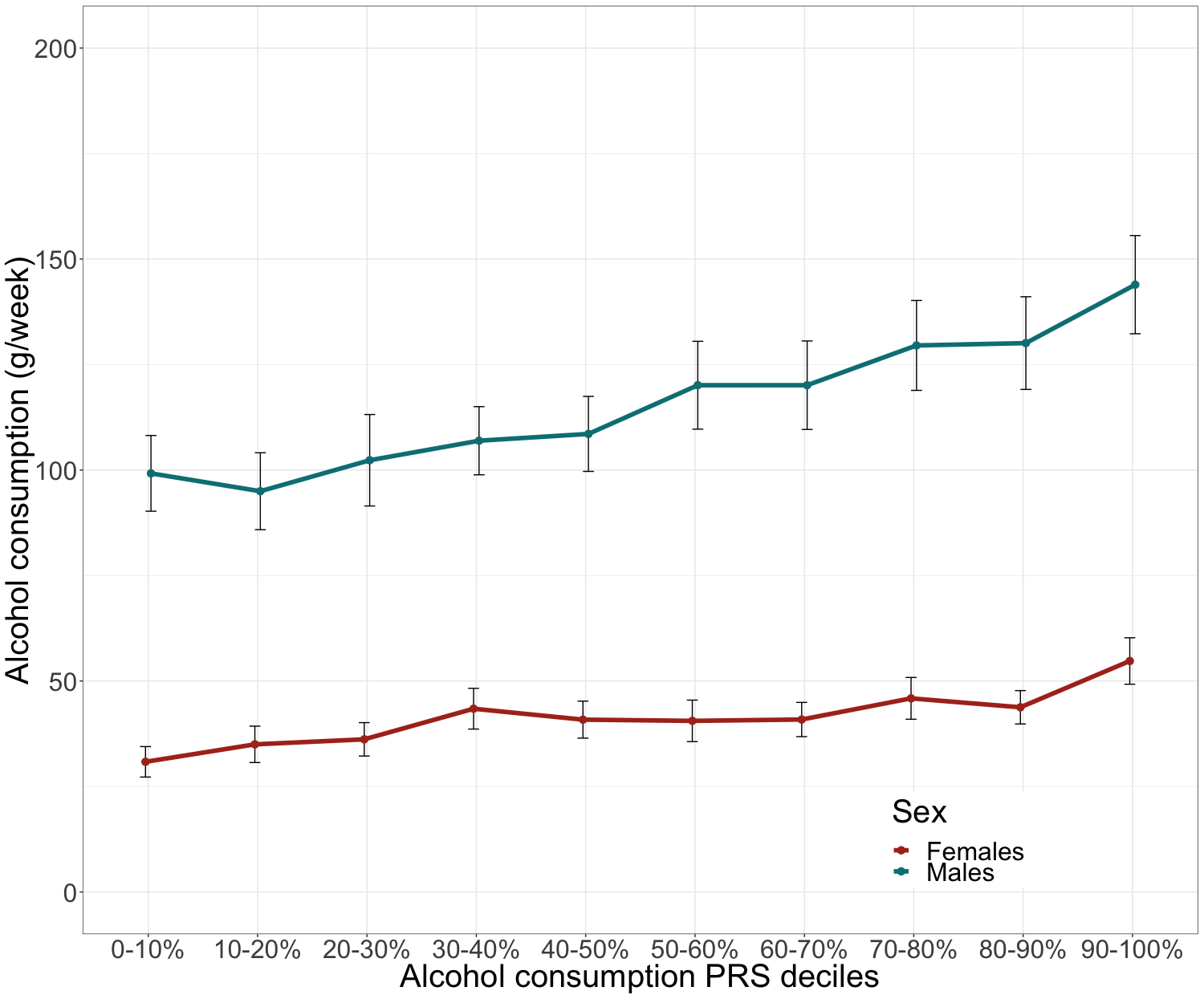
*

**b) Health 2000**

*
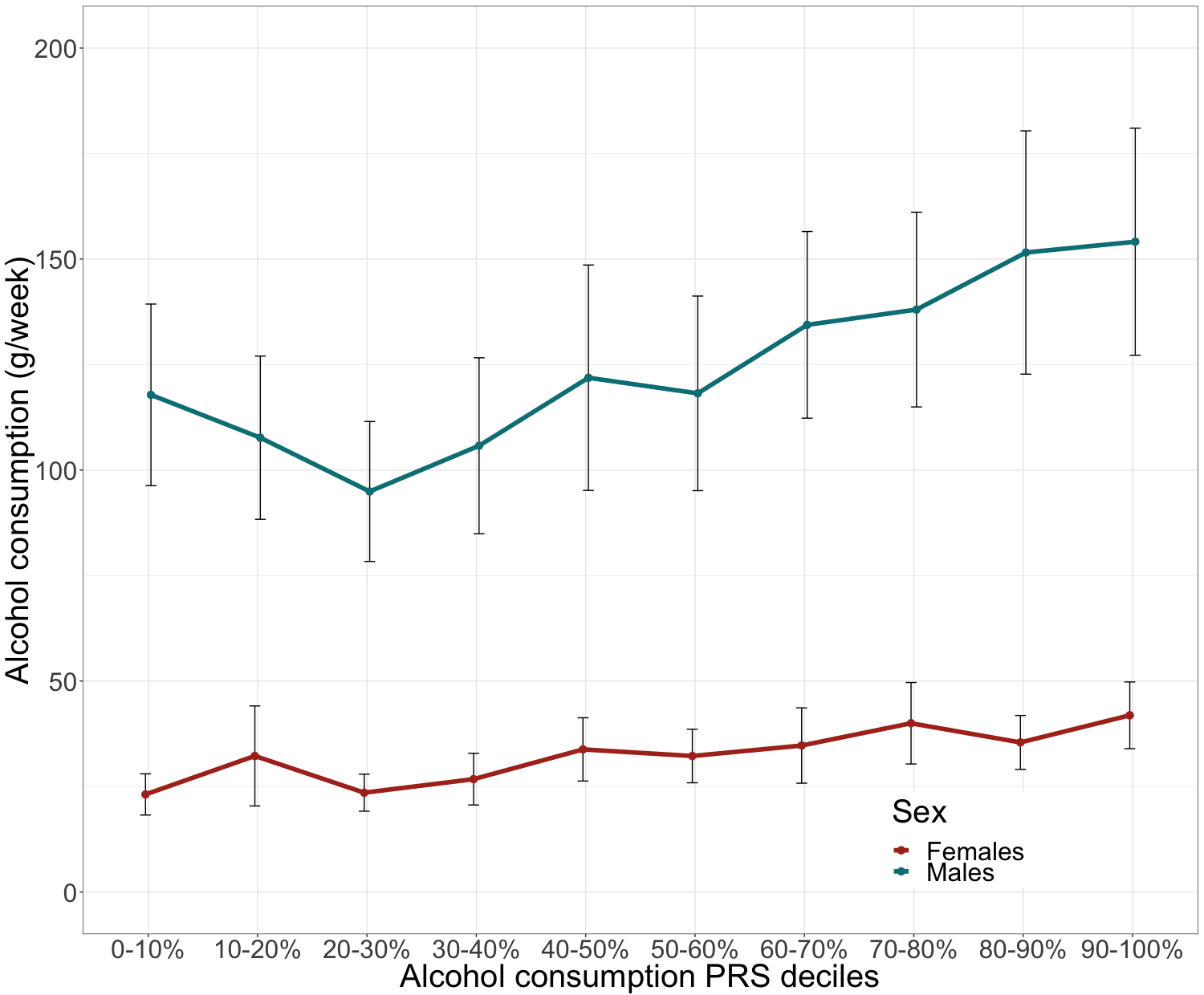
*

**c) Twin Cohort**

*
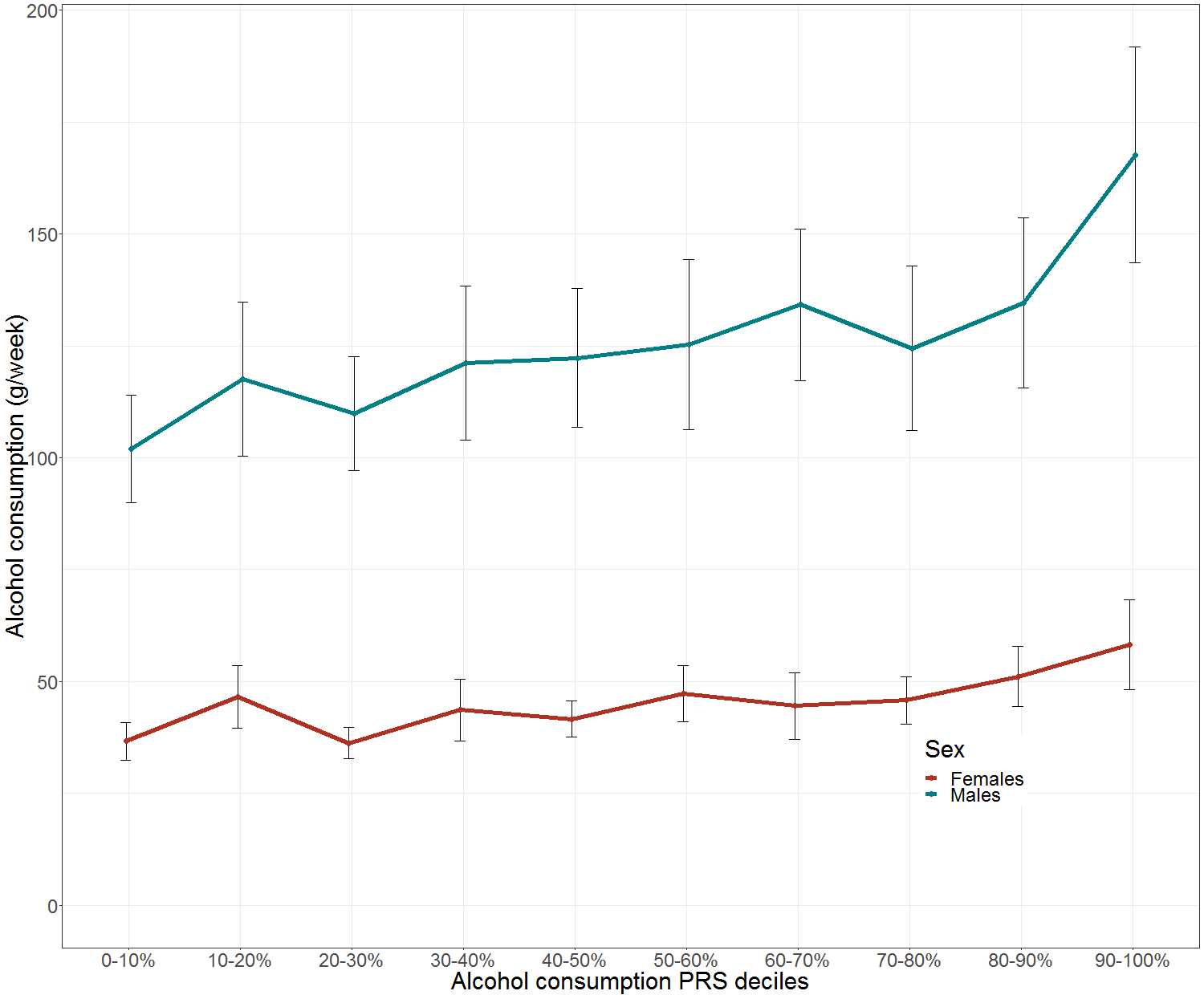
*

## Table S1. The prospective epidemiological and disease-based cohorts and hospital-based samples in FinnGen Data Freeze 2

| Cohort | N |
| --- | --- |
| Auria biobank* | 3,556 |
| Blood Service biobank | 6,271 |
| Borealis biobank* | 1,383 |
| Botnia Family1 | 1,176 |
| Botnia New | 6 |
| Botnia PPP4 | 4,724 |
| Botnia Sib-Helsinki | 428 |
| FinHealth 2017 | 5,938 |
| FINRISK 1992-2012 | 29,774 |
| GeneRISK | 7,090 |
| Health 2000 | 6,672 |
| Helsinki biobank* | 10,410 |
| Health 2011 | 717 |
| Migraine | 7,882 |
| SUPER | 4,420 |
| Diabetes | 6,052 |
| Sum | **96,499** |

*Hospital-based samples

## Table S2. Specific conditions and corresponding ICD/ATC codes that were used in the construction of the combinatory alcohol-related morbidities endpoints

|  | ICD-10 | ICD-9 | ICD-8 | ATC |
| --- | --- | --- | --- | --- |
| Acute alcohol intoxication* | F10.0 |  |  |  |
| Mental and behavioural disorders due to alcohol, excluding non-pathological acute intoxication | F10.1-9 | 291,303,3050A | 291,303 |  |
| Degeneration of nervous system due to alcohol | G31.2 |  |  |  |
| Epileptic seizures related to alcohol | G41.51 |  |  |  |
| Alcohol induced polyneuropathy | G62.1 | 3575A |  |  |
| Alcoholic myopathy | G72.1 |  |  |  |
| Alcoholic cardiomyopathy | I42.6 | 4255 |  |  |
| Maternal care for (suspected) damage to fetus from alcohol | O35.4 |  |  |  |
| Alcoholic gastritis | K29.3 | 5353A |  |  |
| Alcoholic liver disease | K70 | 5710-3 | 5710 |  |
| Acohol-induced acute pancreatitis | K85.2 | 5770D-F |  |  |
| Alcohol-induced chronic pancreatitis | K86.0 | 5771C-D |  |  |
| Fetus and newborn affected by maternal use of alcohol | P04.3 | 7607A |  |  |
| Accidental poisoning by and exposure to alcohol | X45 |  |  |  |
| Guidance and medical advice to a person with alcohol abuse | Z71.4 |  |  |  |
| Alcohol-induced pseudo-Cushing syndrome | E24.4 |  |  |  |
| Toxic effect of ethanol |  |  |  |  |
| Toxic effect of unspecified or or other (than ethanol) alcohols | T51.1-9 | 9801-9 | 9801-9 |  |
| Use of disulfiram, acamprosate or naltrexone |  |  |  | N07BB01, N07BB02, N07BB04 |

**As ICD-10 F10.0 diagnosis can be used only in the absence of AUDs, we included it only in the alcohol-related mortality endpoint definition.*

## Supplementary References

(1) Delaneau O, Zagury J, Marchini J. Improved whole-chromosome phasing for disease and population genetic studies. Nature methods 2013;10(1):5.

(2) Howie B, Fuchsberger C, Stephens M, Marchini J, Abecasis GR. Fast and accurate genotype imputation in genome-wide association studies through pre-phasing. Nat Genet 2012;44(8):955.

(3) Loh P et al. Reference-based phasing using the Haplotype Reference Consortium panel. Nat Genet 2016;48(11):1443.

(4) Das S et al. Next-generation genotype imputation service and methods. Nat Genet 2016;48(10):1284.

(5) McCarthy S et al. A reference panel of 64,976 haplotypes for genotype imputation. Nat Genet 2016;48(10):1279.
